# Characterization of tethered equine chorionic gonadotropin and its deglycosylated mutants by ovulation stimulation in mice

**DOI:** 10.1186/s12896-019-0550-6

**Published:** 2019-08-13

**Authors:** Kwan-Sik Min, Jong-Ju Park, Munkhzaya Byambaragchaa, Myung-Hwa Kang

**Affiliations:** 1 Animal Biotechnology, Graduate School of Future Convergence Technology, Institute of Genetic Engineering, Hankyong National University, Ansung, 17579 Korea; 2 Department of Animal Resource Science, Hankyong National University, Ansung, 17579 Korea; 3 Department of Food Science and Nutrition, Hoseo University, Asan, 31499 Korea

**Keywords:** Rec-eCG, Glycosylated sites, Ovulation rate

## Abstract

**Background:**

To directly assess the biological role of oligosaccharides in recombinant equine chorionic gonadotropin (rec-eCG) functioning, cDNA encoding the full-length eCGβ-subunit was fused with the mature protein part of the α-subunit, and we examined the expression levels of deglycosylated eCG mutants, the ovulation rate for deglycosylated mutants in C57BL/6 mice.

**Results:**

The characterizations of heterodimeric and tethered mutants were studied following their respective secretions in culture medium, molecular weight and ovulation in vivo. Rec-eCG variants containing mutations at glycosylation sites at Asn82 of the α-subunit (eCGβ/αΔ82) and Asn13 of the β-subunit (eCGβΔ13/α) were not efficiently secreted into the culture medium from transfected cells. Western blot analysis revealed that the rec-eCGβ/α proteins have an approximate broad range of molecular weights of 40–46 kDa. Three rec-eCG mutants—a deglycosylated site at Asn56 of the α-subunit (eCGβ/αΔ56), a deletion of the C-terminal region of the β-subunit (eCGβ-D/α), and the double mutant (eCGβ-D/αΔ56)—turned out to have clearly lower (approximately 4–23 kDa) molecular weights. Protein N-glycosydase F (PNGase F) treatment markedly decreased the molecular weight to approximately 2–10 kDa. Normal oocytes were significantly more abundant in the natural eCG–treated group than in mutant rec-eCG–treated groups. In particular, numbers of nonfuntional oocytes were remarkably lower in all rec-eCG groups.

**Conclusions:**

Our results indicate that the ovulation rates of oocytes are not affected by the deglycosylated rec-eCGβ/α mutant proteins. There are around 20% non-functional oocytes with natural eCG and only 2% with the rec-eCGs tested. These results provide insight into the molecular mechanisms underlying the production of rec-eCG hormones with excellent bioactivity in vivo.

**Electronic supplementary material:**

The online version of this article (10.1186/s12896-019-0550-6) contains supplementary material, which is available to authorized users.

## Background

Chorionic gonadotropin (CG) is a placental hormone that maintains the corpus luteum (CL) during pregnancy [1]. CG exist only in primates and equidaes and not in the other mammals. Gonadotropins are heterodimeric glycoprotein hormones, consisting of dissimilar α- and β-subunits that are noncovalently complexed [2]. The α-subunit of the glycoproteins is common for luteinizing hormone (LH), follicle-stimulating hormone (FSH), and thyroid-stimulating hormone (TSH) in a given species [3].

Equine CG (eCG) is a unique member of the gonadotropin family because it exhibits both LH- and FSH-like activities in non-equid species [4, 5]. The β-subunits of eCG and equine LH (eLH), being translated from the same gene, have an identical primary structure [6, 7]. Thus, eCG may be an ideal model for studying the structure-function relations of gonadotropins because it possesses properties of both its pituitary and placental counterparts [8, 9]. The difference between eCG and eLH lies in the structure of their carbohydrates, which are sialylated and sulfated in LH but only sialylated in CG [10, 11]. eCG is secreted from binucleate trophoblastic cells in endometrial cups, into maternal blood plasma during the first half of equine gestation. These cells get detached from the chorionic girdle of the conceptus between days 37 and 120 of pregnancy [12–14].

eCG administration has also been associated with an increase in ovulation rate [15], particularly in early-postpartum cows [16]. The standard dose of eCG required to promote single ovulation generally ranges between 200 and 1000 IU, whereas the dose necessary to induce superovulation is approximately 2500 IU [17]. Similarly, eCG administration to sheep has been demonstrated to significantly increase the level of proteins FSH receptor (FSHR) and gonadotropin-releasing hormone receptor (GnRHR) secreted by oocytes [18]. Thus, eCG enhances maturation and stimulates FSHR, LHR, and GnRHR expression. In some studies on the secretion and activity of recombinant eCG (rec-eCG) in mammalian cells, deletion of carboxy-terminal peptides (CTPs) from dimeric eCG induced a 50% decrease in the secretion of the truncated hormone as compared to the wildtype [19]. FSH activity strongly depends on amino acid residues (aa) 102–104 of the eCGβ-subunit [20], and correct folding and FSH activity are conferred by aa 104–109 of the eCGβ-subunit [9].

Some studies have revealed that glycosylation of α-subunit position 52 in human FSH (hFSH) [21], human chorionic gonadotropin (hCG) [22], and hTSH [23] is important for signal transduction, because cAMP or steroid formation is not stimulated, while the binding activity with FSHR was enhanced by 2- to 3-fold [24]. Thus, receptor binding and signal transduction are dissociable functions involving different sites on the FSH glycoprotein. Various studies suggest that CTPs of the deletion-containing (aa 115–145) hCGβ-subunit [25, 26], and deletion-containing (aa 122–145) hCGβ [27] are not important for receptor binding or in vitro signal transduction. Nontheless, the truncated form of hCGβ lacking aa 101–145 is the shortest form of the subunit known to retain biological activity [28]. In eLH, the deletion containing (aa 121–149) β-subunit is incapable of subunit association and receptor binding [29]. These results are consistent with findings of our previous research into the effects of deglycosylated rec-eCG mutants on estradiol and progesterone stimulation of rat granulosa cells and Leydig cells [5, 30].

To investigate the functional contribution of oligosaccharides in rec-eCGs and of carboxy-terminal extension (aa 114–149) of the eCGβ-subunit, we created a total of 20 expression vectors encoding 11 heterodimeric eCGs and nine tethered eCGs. We produced rec-eCGα/β and eCGβ/α proteins in CHO-K1 cells, characterized these proteins’ biological activities in vivo. Our results indicate that the loss of glycosylation at site Asn82 of the α-subunit and at Asn13 of the β-subunit plays a pivotal role in secretion into the culture medium of CHO-K1 mammalian cells. Deglycosylated rec-eCGs showed a high oocyte ovulation rate in vivo.

## Results

### Quantities of heterodimeric rec-eCGα/β and tethered rec-eCGβ/α proteins

**Fig. 1:**
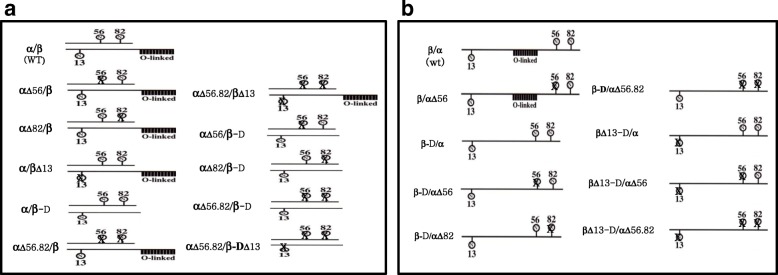
A schematic diagram of rec-eCGα/β mutants (**a**) and rec-eCGβ/α mutants (**b**). The wild-type protein and mutants with changed N- and O-linked oligosaccharide sites on eCG are shown. The Asn56 and 82 codons in the α-subunit were replaced by Gln or the CTP carrying O-linked oligosaccharides in the eCGβ-subunit was deleted by PCR mediated site-directed mutagenesis. The circle “N” denotes an N-linked oligosaccharide, “X” indicates the absence of an oligosaccharide, and “O-linked” represents an O-linked oligosaccharide. A total of 20 expression vectors (for 11 heterodimeric eCGs and nine tethered eCGs) were constructed (plasmids encoding heterodimeric eCGs were designated as pAB-eCGα/β, pAB-αΔ56/β, pAB-αΔ82/β, pAB-α/β Δ13, pAB-α/β-D, pAB-αΔ56,82/β, pAB-αΔ56/βΔ13, pAB-αΔ56/β-D, pAB-αΔ82/β-D, pAB-αΔ56.82/β-D, and pAB-αΔ56.82/βΔ13; plasmids encoding tethered eCGs were designated as pcDNA3-eCGβ/α, pcDNA3-β/αΔ56, pcDNA3-β-D/α, pcDNA3-β-D/αΔ56, pcDNA3-β-D/αΔ82, pcDNA3-β-D/αΔ56.82, pcDNA3-βΔ13-D/α, pcDNA3-βΔ13-D/αΔ56, and pcDNA3-βΔ13-D/αΔ56.82)

Oligosaccharide-directed mutagenesis was carried out examine the functional importance of oligosaccharides in eCG bioactivity. The eCGα-subunit contains two N-linked glycosylation sites at aa positions 56 and 82. The eCGβ-subunit contains one N-linked glycosylation site at aa position 13 and approximately 11 O-linked glycosylation sites in the C-terminal region. Thus, we constructed 20 expression vectors encoding 11 heterodimeric eCG mutants and nine tethered eCG mutants, respectively (Fig. 1).

**Fig. 2:**
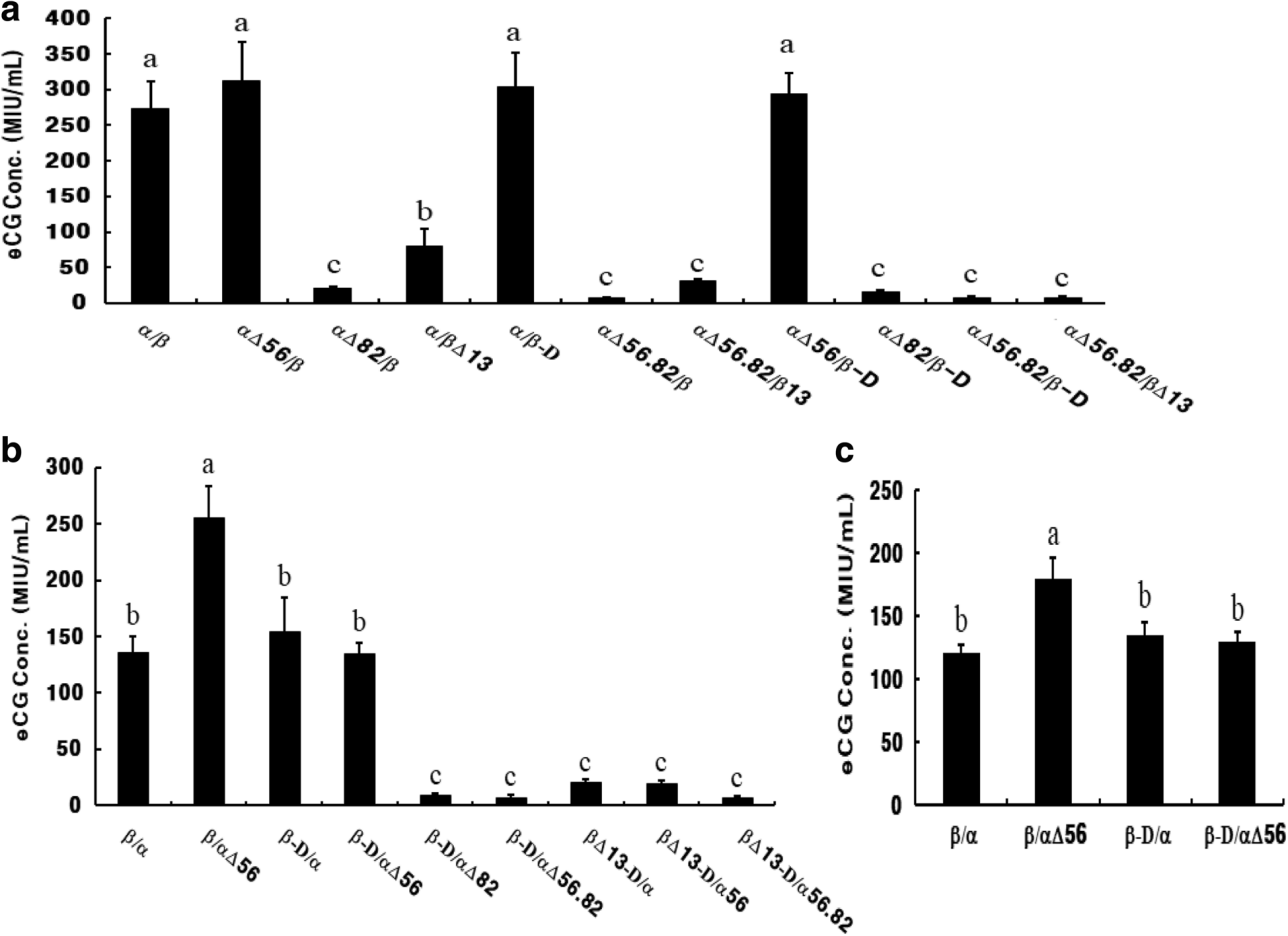
Quantitative analysis of rec-eCG mutants and myc-tagged rec-eCG mutants by ELISA after transient transfection into CHO-K1 cells. **a** Quantities of dimeric rec-eCG mutants were measured by an ELISA. **b** Tethered rec-eCG mutants were detected. **c** Quantities of *myc*-tagged rec-eCG mutants were analyzed by an ELISA. The *myc*-tag (Glu-Gln-Lys-Leu-Ile-Ser-Glu-Glu-Asp-Leu) was added between the first and second amino acid residue of the β-subunit of the mature eCG protein. Values with different superscripts are significantly different (*p* < 0.05)

The expression vectors were transiently transfected into cells and the culture supernatant was collected at 72 h after transfection. Stable colonies resistant to G418 were selected, and rec-eCGs secreted into the serum-free medium were collected and concentrated. The rec-eCGs were quantified by a pregnant mare serum gonadotropin (PMSG) enzyme-linked immunosorbent assay (PMSG ELISA). Although the rec-eCG protein was detected at a low concentration when it contained Asn13 in the eCGβ-subunit, the protein (deglycosylated mutant) was nearly undetectable when it contained a mutation of Asn82 in the eCGα-subunit and a mutation of Asn13 in the eCGβ-subunit (Fig. 2a, b). Expression levels of the heterodimeric rec-eCGs (α/β, αΔ56/β, α/β-D, and αΔ56/β-D) were 273 ± 38.1, 313 ± 53.5, 304 ± 47.1, and 294 ± 28.3 ng/mL, respectively. Expression levels of heterodimeric αΔ82/β and α/βΔ13 were 21 ± 2.5 and 80 ± 23.8 ng/mL, respectively. These levels were slightly lower for the tethered rec-eCGs. The secreted quantities of rec-eCG β/α, β/αΔ56, β-D/α, and β-D/αΔ56 were 136 ± 13.7, 255 ± 28.3, 154 ± 30.3, and 135 ± 8.8 ng/mL, respectively. Nevertheless, the secretion pattern was the same as that of dimeric eCG. These results indicate that a loss of glycosylation at site Asn82 of the α-subunit and at Asn13 of the β-subunit plays a pivotal role in secretion into the culture medium from CHO-K1 cells. On the other hand, deletion of the eCGβ-subunit CTP did not affect the secretion into the culture medium from CHO-K1 cells. We also compared the expression between mutants eCGβ/α and *myc* eCGβ/α; the latter contains a *myc*tag (Fig. 2c). The expression levles of these proteins were nearly the same.

### Western blot analysis of rec-eCGβ/α glycosylation mutants

**Fig. 3:**
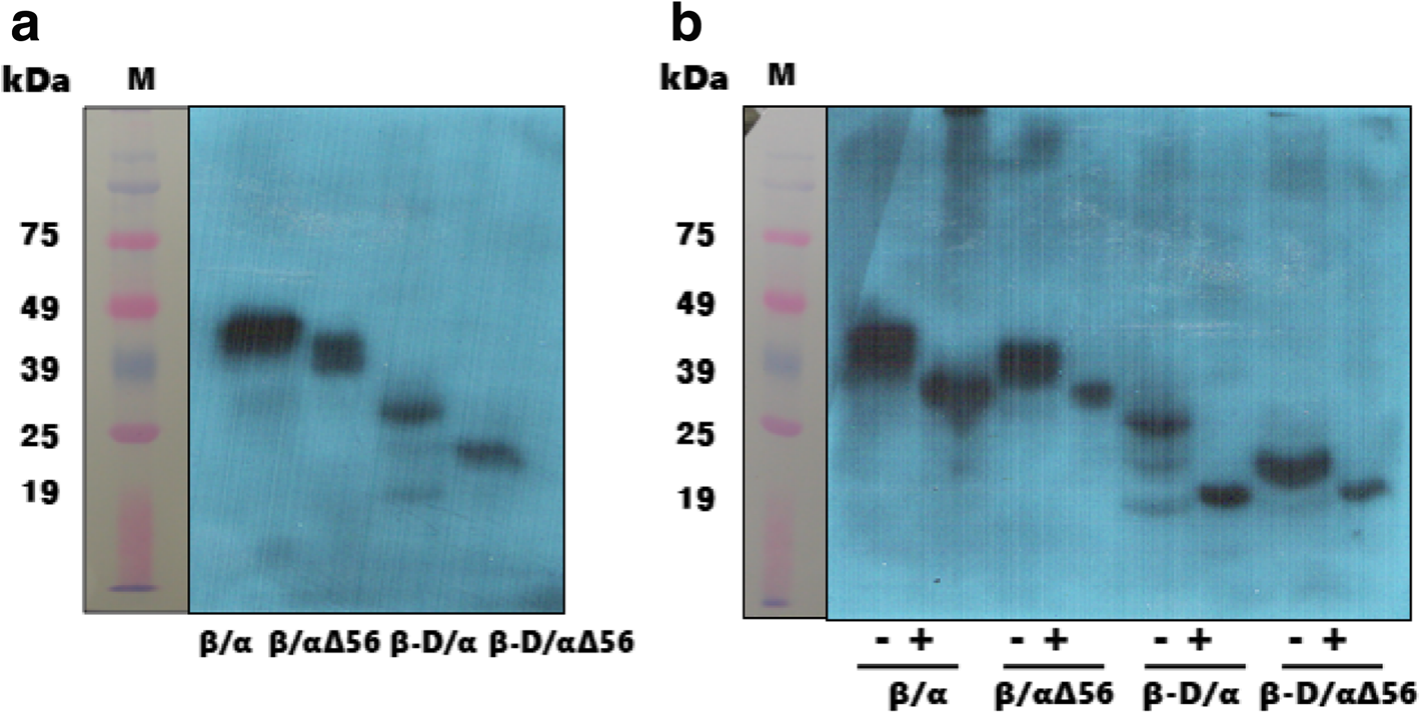
Western blot analysis of tethered rec-eCG mutants. **a** Rec-eCG mutants were expressed in CHO-K1 cells. The proteins in conditioned media were collected, separated by SDS-PAGE, and transferred to a blotting membrane. The proteins were detected with antibodies against the *myc*-tag. **b** Proteins for western blotting were also treated with N-GlycosidaseF. Lane 1: β/α, Lane 2: β/αΔ56, Lane 3: β-D/α, Lane 4: β-D/αΔ56,-: not treated, +: treated with N-Glycosidase-F

Next, we studied the tethered rec-eCG mutants to determine molecular weight and biological activity. By western blot analysis, we found an approximate range of molecular weight of 40–46 kDa for rec-eCGβ/α, as shown in Fig. 3a. Rec-eCGβ/αΔ56, without N-linked oligosaccharides at Asn56 of the α-subunit, was reduced to approximately 37–42 kDa, a decrease of ~ 3–4 kDa. Rec-eCGβ-D/α, with a deletiion of 35 aa including the O-glycosylated CTP of the β-subunit, remarkably diminished to approximately 27 kDa. The molecular weight of the double mutants (rec-eCGβ-D/αΔ56) decreased to ~ 23 kDa. After deglycosylation treatment with protein N-glycosydase F (PNGase F), the molecular weights of rec-eCGβ/α, β/αΔ56, β-D/α, and β-D/αΔ56 decreased to approximately 30–36, 33, 19, and 19 kDa, respectively (Fig. 3b). These results indicated that oligosaccharides were largely modified in rec-eCGs produced in CHO-K1 cells and in the mutant proteins that were deglycosylated in the N-linked and O-linked regions of eCGα- and β-subunits, confirming the loss of oligosaccharide chains.

### In vivo biological activity of rec-eCG mutants

To test the in vivo activity of natural eCG and of rec-eCG derivatives, we examined oocyte ovulation. The numbers of normal ovulated oocytes in the natural-eCG group were 22.6 ± 2.9 as presented in Table 1. By contrast, the other groups (β/α, β/αΔ56, β-Dα, and β-D/αΔ56), which were injected with rec-eCG proteins produced 13.5 ± 1.3, 14.8 ± 1.2, 14 ± 1.8, and 15 ± 0.6 oocytes, respectively. The number of normal oocytes after treatment with natural eCG was higher relative to the rec-eCG groups. Nonetheless, there were no differences among the rec-eCG groups. We also examined the nonfunctional oocytes which were detected in the natural-eCG–treated group (Additional file 1: Figure S1). Nearly the same numbers of nonfunctional oocytes were found in the rec-eCG–treated groups. These results meant that the rec-eCG proteins produced in this study had full activity and were capable of inducing ovulation.

**Table 1:** Superovulation of mouse oocytes between natural-eCG and tethered rec-eCGβ/α deglycosylated mutants

	No. Oocytes		
	Functional	Nonfunctional	Nonfunctional relative to total oocytes (%)
PMSG (*n* = 9)	22.6 ± 2.9A	4.78 ± 0.5a	21.2
rec-eCGβ/α (*n* = 6)	13.5 ± 1.3B	0.33 ± 0.2b	2.4
rec-eCGβ/αΔ56 (n = 6)	14.8 ± 1.2B	0b	0
rec-eCGβ-D/α (n = 6)	14 .0 ± 1.8B	0.33 ± 0.3b	2.4
rec-eCGβ-D/αΔ56 (n = 6)	15.0 ± 0.6B	0b	0

The mice were superovulated by injection of 10 IU of PMSG or rec-eCGs and then 10 IU hCG after 48 h, and ovulated oocytes were collected into an oviduct ampulla after 13 h as described in Methods. 1 IU was assumed to be 100 ng according to the conversion factor of the suggested assay protocol corresponding PMSG by standard curve. The ovulated oocytes were determined under microscope as shown in Additional file 1: Figure S1. The percentage of nonfunctional relative to total oocytes was around 20% with natural eCG and only around 2% with rec-eCGs used

Values are expressed as mean ± SEM for at least three independent experiments

A-B, a-bValues with different superscripts are significantly different (*p* < 0.05)

## Discussion

We examined the biological roles of N-linked oligosaccharides and the O-linked CTP of eCG, as well as ovulation induction, in vivo by means of rec-eCG generated by site-directed mutagenesis. Another study [31] on rec-hCG suggest that mutagenesis of Asn at position 52, which is linked to an oligosaccharide, does not affect secretion of the mutant subunit. In contrast, the loss of the oligosaccharide at position 78 was found to cause the mutant subunit to be degraded quickly, and < 20% was secreted. The molecular weights of secreted wild-type hCG and mutant protein hCGαΔ52/β turned out to be 28 and 22 kDa, respectively. Furthermore, some researchers reported that the absence of both N-linked units (Asn13 and Asn30) in the hCGβ-subunit slows secretion by 2–2.4-fold [32]. As for rec-hTSH, wild-type TSH has a molecular weight of ~ 38 kD, whereas the TSH single-site mutant (TSHα1 or TSHα2) and TSH double mutant (TSHα1 + α2) are approximately 35 and 31 kDa, respectively [23]. Our results are consistent with those of other studies, suggesting that secretion into the culture medium is controlled by the specific glycosylation sites (Asn82 in the α-subunit and Asn13 in the β-subunit). Thus, the Asn-linked oligosaccharides of eCGα- and β-subunits may have site-specific functions with respect to secretion. The secreted single-chain eCG in COS-7 cells is detectable as a doublet of 46 and 44 kDa [9]. We confirmed that the loss of oligosaccharide chains greatly decreases the molecular weight of the deglycosylated N-linked and O-linked rec-eCGα/β mutants.

Regarding in vitro biological activity, some studies have revealed that glycosylation of Asn52 of the hFSHα-subunit results in a significant decrease in potency (to 26% of the wild-type level) [21] of signal transduction, but binding activity is enhanced 2- to 3-fold [24]. Thus, they proposed that receptor binding and signal transduction are dissociable functions involving different sites on the FSH glycoprotein. The α-subunit Asn52 oligosaccharide has a disproportionate role in signal transduction, and the amino acid sequence near β-subunit Asn24 functions in both binding and signal transduction [33]. In hCG, the Asn52 oligosaccharide has been found to be important for signal transduction; without this oligosaccharide, cAMP stimulation and steroid formation fail [22]. Deletion of oligosaccharide units from either site 1 (Asn52) or site 2 (Asn78) of the α-subunit increased the biological activity of the dimer in that study by approximately 30%. Nevertheless, carbohydrate unit at both sites (Asn52 and Asn78) of the hTSH α-subunit significantly reduces cAMP formation (by ~ 70%) and T3 secretion (by ~ 40%) as compared to wild-type hTSH [23]. Furthermore, the CTP of hCGβ does not interact directly with the choriogonadotropin hormone receptor (LH-CGR) complex and apparently does not influence the tertiary structure or folding of the β-subunit [22, 26, 31]. Besides, we have found that Asn56 of the eCGα-subunit does not enhance agonist stimulation internalization through rat LH/CGR (rLH/CGR) and rFSHR [34].

Various studies indicate that rec-eCG yield the formation and secretion of stable heterodimeric eCG in infected Sf9 cells [35] and COS-7 cells [36], and that rec-eCG has the same thermal stability as natural pituitary LH [37]. O-linked oligosaccharides of the CTP in the hCGβ-subunit play only a minor role in receptor binding and signal transduction in vitro. By contrast, these oligosaccharides are critical for the in vivo biological response, suggesting that truncated hCG results in significantly fewer ovulated oocytes at doses of 70 and 210 ng [22]. We have previously reported that heterodimeric rec-eCGα/β exert LH- and FSH-like activities similar to those of native eCG in in vitro bioassays of primary cultured rat Leydig and granulosa cells, respectively [5], and the same is true for tethered eCGβ/α [30]. We have also shown previously that rec-eCG of nonequid species has both LH- and FSH-like activities in in vitro experiments on cells expressing rLH/CGR and rFSHR [34] and in terms of receptor function [38]. Nevertheless, no studies have examined the ovulation rate in mice under the influence of rec-eCG derivatives. Here, we suggest that the ovulation rates in mice may not be affected by the deglycosylating mutations. Nevertheless, it is impossible to compare potencies by means of only one dose, 10 IU injection. Thus, biological activity of rec-eCG derivatives slightly differs in vitro and in vivo.

## Conclusions

Thus, secretion into the culture medium in rec-eCGβ/α is controlled by specific glycosylation sites: Asn82 of the α-subunit and Asn13 of the β-subunit. The Asn-linked oligosaccharides of eCGα- and β-subunits have site-specific functions with respect to secretion. Besides, we confirmed by removal of oligosaccharide chains that the molecular weight was decreased by deglycosylation of the N-linked and O-linked eCGα- and β-subunits. The nonfunctional oocytes were around 20% in the natural eCG-treated mice. However there are only around 2% with the rec-eCG derivatives tested. We also suggest that the differences between rec-eCG and natural-eCG could rather be due to the nature of the injected hormone. Therefore, rec-eCGβ/α derivatives have a good potential to produce functional oocytes with a much smaller number nonfunctional oocytes.

## Methods

### Materials

The oligonucleotides employed in this study were synthesized by Genotech (Daejon, Korea). The following reagents and materials were also used: restriction enzymes, polymerase chain reaction reagents, and the DNA ligation kit were purchased from Takara (Tokyo, Japan). CHO-K1 cells were obtained from the Japanese Cancer Research Resources Bank (Tokyo, Japan). Ham’s F-12 medium, Opti-MEM I, serum-free CHO-S-SFM II, geneticin, and Lipofectamine 2000 were bought from Gibco BRL (Grand Island, NY, USA), and fetal bovine serum from Hyclone Laboratories (Logan, UT, USA). The QIAprep-Spin plasmid kit and RNeasy columns were acquired from QIAGEN, Inc. (Hilden, Germany), whereas the Pro-Prep™ protein extraction solution from Intron Biotechnology (Seoul, Korea). The Lumi-Light western blot kit was bought from Roche (Basel, Switzerland), and the pcDNA3 mammalian expression vector, PNGase F, and TRIzol reagent from Invitrogen (Carlsbad, CA, USA). The PMSG ELISA kit was purchased from DRG International, Inc. (Mountainside, NJ, USA), Centriplus Centrifugal Filter Devices from Amicon Bio separations (Merck, Billerica, MA, USA), and an anti-*myc* antibody from Santa Cruz Biotechnology (Dallas, Texas, USA). A peroxidase-conjugated anti-mouse IgG antibody was acquired from Bio-Rad (Hercules, CA, USA), whereas pregnant-mare serum gonadotropin (eCG) from Sigma-Aldrich Corp. (St. Louis, MO, USA), as were all other reagents. All experimental designs and procedures were in compliance with the approved Guidelines for Animal Experiments of Hankyong National University, Korea, and were approved by the Animal Care and Use Committee of Hankyong National University, Korea (Approval ID: 2015–8).

### Construction of heterodimeric eCGα/β and tethered eCGβ/α

The heterodimeric eCGα/β and its mutants were created using the pABWN vector as previously reported [5]. Additionally, cDNA encoding the tethered rec-eCGβ/α was inserted into the pcDNA3 vector and served as a template to construct mutants in which Asn (codon AAC) of the glycosylation site was substituted with Ala (codon CAG) or the CTP was deleted in the β-subunit as reported elsewhere [30]. The same method was used to add a *myc*tag (Glu-Gln-Lys-Leu-Ile-Ser-Glu-Glu-Asp-Leu) between the first and second amino acid residues of the β-subunit of the mature eCG protein [38]. Site-directed mutagenesis was performed with three primers in a single-step PCR [5]. The primers employed in the experiment are summarized in Table 2). The first PCR was carried out with primers 2 and 3. DNA fragments were spliced and subjected to a second PCR involving primers 1 and 12 to generate tethered eCGs. The schematic diagrams of heterodimeric rec-eCGα/β and tethered rec-eCGβ/α mutants are depicted in Fig. 1.

**Table 2:** List of primers used for the construction of tethered eCG mutants

	Primer name	Location	Primer Sequence
1	eCG*β/α β*-subnuit	-	5′- T**GAATTC**ACCATGGAGACGGTCCAG -3′ ***Eco R I*** **site**
2	*myc* eCG*β/α* reverse	-	5′- TCCATCAGGAAAAGAAGTCTTTATTGG -3′
3	*myc* eCG*β/α* forward	-	5′- ATAAAGACTTCTTTTCCTTGATGGAGAG -3′
4	eCG*β/α*Δ*56* reverse	*α* 56	5′- TGAGGTGATCTGCTTTGGGACCAACAT -3′
5	eCG*β/α***Δ***56* forward	*α* 56	5′- ATGTTGGTCCCAAAGCAGATCACCTCA -3′
6	eCG*β/α***Δ***82* reverse	*α* 82	5′- GGCCTCCTTCTCAGCGGCCAGAGTGGCGTTGAT -3′
7	eCG*β/α***Δ***82* forward	*α* 82	5′- ATCAACGCCACTCTGGCCGCTGAGAAGGAGGCC -3′
8	eCG*β***Δ***13/α* reverse	*β* 13	5′- AGCAGCCAGAGTGGCGTTGATGGGCCGGCACAG -3′
9	eCG*β***Δ***13/α* forward	*β* 13	5′- CTGTGCCGGCCCATCAACGCCACTCTGGCTGCT -3′
10	eCG*β-D/α* reverse	*β* : 121-149	5′- TCCATCAGGAAAGGCCTGGGGGGCACAGGC -3′
11	eCG*β-D/α* forward	*β* : 121-149	5´- GCCCCCCAGGCCTTTCCTCATGGAGAGTTT -3´
12	eCG*β/α α*-subunit	-	5′- CC**GTCGAC**TTTAAATCTTGTGGTGATAGCA -3′ ***Sal I*** **site**

We mutated Asn to Gln at each glycosylation site (Asn56, Asn82, Asn13, deletion of the β-subunit CTP region with one site and a combination). These fragments were digested with *EcoR*I and *Sal*I and ligated into eukaryotic expression vector pcDNA3. The plasmid DNAs were purified and sequenced in both directions by automated DNA sequencing to ensure that the correct mutations were introduced. A total of 20 expression vectors (11 heterodimeric eCGs and nine tethered eCGs) were constructed (plasmids expressing heterodimeric eCGs were designated as pAB-eCGα/β, pAB-αΔ56/β, pAB-αΔ82/β, pAB-α/βΔ13, pAB-α/β-D, pAB-αΔ56,82/β, pAB-αΔ56/βΔ13, pAB-αΔ56/β-D, pAB-αΔ82/β-D, pAB-αΔ56.82/β-D, and pAB-αΔ56.82/βΔ13; plasmids expressing tethered eCGs were designated as pcDNA3-eCGβ/α, pcDNA3-β/αΔ56, pcDNA3-β-D/α, pcDNA3-β-D/αΔ56, pcDNA3-β-D/αΔ82, pcDNA3-β-D/αΔ56.82, pcDNA3-βΔ13-D/α, pcDNA3-βΔ13-D/αΔ56, and pcDNA3-βΔ13-D/αΔ56.82) as previously reported [5, 30, 34].

### Cell culture and functional expression

Expression vectors were transfected into CHO-K1 cells by the liposome transfection method as described in ref. [30]. The transfected cells were cultured for 48 h in a serum-free medium (CHO-S-SFM-II) and then harvested and centrifuged at 100,000×*g* for 10 min. The supernatant was collected and stored at − 20 °C until the assay. Six to eight pools of stably transfected cells were selected by incubating the cells in a growth medium [Ham’s F12 medium with 10% of fetal calf serum, penicillin (100 IU/mL), streptomycin (100 μg/mL), glutamine (2 mM), and G418 (800 μg/mL)] for 2–3 weeks after transfection, as previously reported [30]. The culture medium was centrifuged at 100,000×*g* for 10 min to remove cell debris. The supernatant was collected and concentrated in an Amicon Stirred cell concentrator and stored at − 20 °C until the assay.

### Quantification of rec-eCG proteins

The plasmids encoding dimeric eCG and single-chain eCG were transiently transfected into CHO-K1 cells, and the culture media were collected at 72 h after transfection, and rec-eCG was quantified with the PMSG ELISA kit (DRG Diagnostics). Briefly, the collected medium was centrifuged to remove cell debris at 5000 rpm for 3 min. The supernatant was collected and stored at − 20 °C until analysis. The PMSG standard and rec-eCG samples (100 μL) were dispensed into wells of a plate coated with a monoclonal antibody against a unique antigenic site on the eCG molecule. The plate was incubated for 60 min at room temperature without agitation. Then, we briskly shook out the contents of the wells and rinsed them three times with distilled water. After that, 100 μL of a anti-PMSG antibody conjugated to horseradish peroxidase was added into each well and incubated for 60 min at room temperature without agitation. The plate wells were rinsed five times, and residual water droplets were removed. A substrate solution (100 μL) was next added and incubated for 30 min at room temperature. Finally, 50 μL a stop solution was added to stop the enzymatic reaction. The absorbance of the product solution was read at 450 nm on a micro titer plate reader. The average absorbance of each standard was plotted against its corresponding concentration in a linear–log graph. We calculated the average absorbance of each sample to determine the corresponding PMSG value via simple interpolation by means of this standard curve. Finally, 1 IU was assumed to be 100 ng according to the conversion factor of the suggested assay protocol.

### Detection of rec-eCGs by western blotting and enzymatic digestion of N-linked oligosaccharides

For western blot analysis, samples of a concentrated medium were subjected to electrophores is under reducing conditions in sodium dodecyl sulfate (SDS) 12.5% polyacrylamide gels by the Laemmli method [39]. After SDS polyacrylamide gel electrophoresis (PAGE), the proteins were transferred to a polyvinylidene difluoride membrane (0.2 μm) at 100 V for 2 h in a Mini Trans-Blot Electrophoretic Transfer cell. After blotting, the membrane was blocked with a 1% blocking reagent for 1 h, and the monoclonal anti-*myc* antibody (1:5,000) was added for 2 h incubation. Next, the blot was washed to remove the unbound antibody, incubated with a secondary antibody (peroxidase-conjugated anti-mouse IgG antibody 37.5 μL/15 mL of the blocking solution) for 30 min, and washed. The membrane was then incubated for 5 min with 2 mL of the Lumi-Light substrate solution and placed with its protein side up on a plastic wrap. The membrane was covered with a second piece of the plastic wrap, and an X- ray film was exposed to the membrane for 1–10 min. The tethered rec-eCG protein was analyzed regarding removal of added glycans by *N-*glycosylation enzyme. To remove all N-linked glycans, rec-eCG was incubated for 24 h at 37 °C with PNGase F [2 μL of the enzyme (2.5 U/mL) per 30 μL of sample+ 8 μL of 5× reaction buffer]. The reaction was stopped by boiling for 10 min, and the samples were analyzed by SDS-PAGE and western blotting.

### In vivo biopotency

This property of rec-eCGβ/α proteins was evaluated by determining the ovulated-oocyte number. Oocytes were collected from 8-week-old B6D2F1 female mice. The mice were superovulated by injection of 10 IU of PMSG or rec-eCGs and then 10 IU hCG after 48 h, and ovulated oocytes were collected into an oviduct ampulla after 13 h. Cumulus cells were removed from the collected oocytes by means of 0.3% hyaluronidase. The oocytes were analyzed for several characteristics (e.g., cytoplasmic membrane, first polar body, and morphological features).

### Data and statistical analysis

Values are given as mean ± SEM. One-way ANOVA with Tukey’s multiple-comparison test was conducted to compare the results between samples. In figures, superscripts indicate significant differences from a group (*p* < 0.05).

## Additional file

Additional file 1: **Figure S1.** Functional and nonfunctional oocytes. (PPT 289 kb)

## Data Availability

The datasets used and analyzed in the current study are available from the corresponding author on reasonable request.

## Abbreviations

CL: Corpus luteum
CTP: Carboxy-terminal peptides
FSH: Follicle-stimulating hormone
FSHR: FSH receptor
GnRHR: Gonadotropin releasing hormone receptor
LH: Luteinizing hormone
PMSG: Pregnant mare serum gonadotropin
rec-eCG: Recombinant equine chorionic gonadotropin
SDS-PAGE: Sodium dodecyl sulfate-polyacrylamide gel electrophoresis
TSH: Thyroid-stimulating hormone
