# Supplementary material for: Characterization of tethered equine chorionic gonadotropin and its deglycosylated mutants by ovulation stimulation in mice
Source: BMC Biotechnol. 2019 Aug 13;19:60. doi: 10.1186/s12896-019-0550-6 (PMC6692925; doi:10.1186/s12896-019-0550-6)
Supplement: Supplementary file 1 — Figure S1. Functional and nonfunctional oocytes. (PPT 289 kb) [file 12896_2019_550_MOESM1_ESM.ppt]

## Slide 1
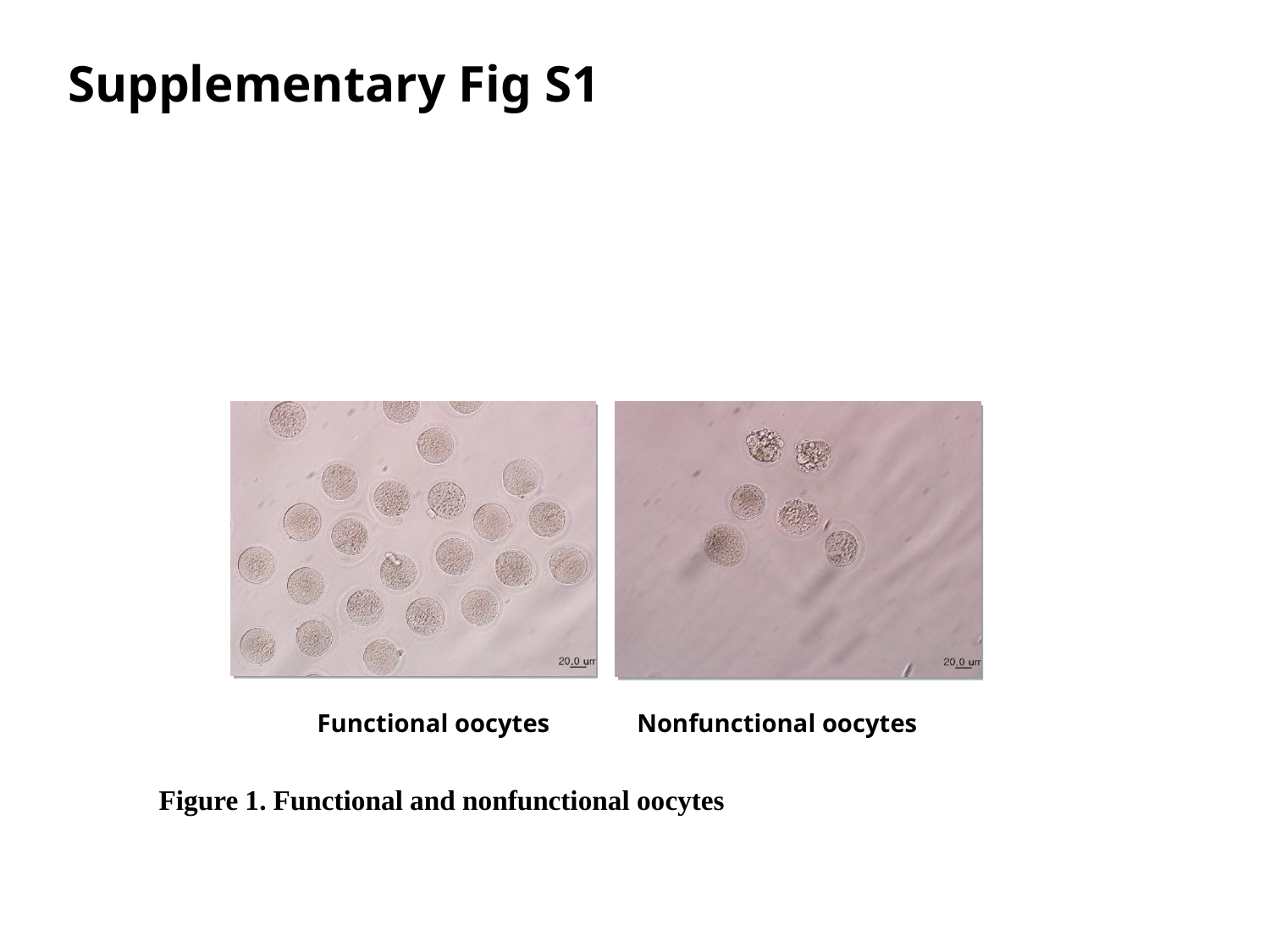

Supplementary Fig S1
Functional oocytes
Nonfunctional oocytes
Figure 1. Functional and nonfunctional oocytes
